# Diabetes

**Published:** 1886-06

**Authors:** 


### DIABETES.

Diabetes is an extremely dangerous and generally fatal disease, the principal symptom of which is an amazing discharge of an excessive quantity of pale urine It is invariably preceded by a general derangement of health, the causes of which are not suspected; this is at length accompanied by pains about the loins, and a daily increasing discharge of morbid urine strongly impregnated with sugar, excessive thirst, great restlessness in the night from these causes, and extreme personal misery. As the fatal termination approaches the emaciation becomes pitable, the strength fails, the gums become red and swollen, and bleed with the slightest pressure, the tongue is foul, with red edges, the appetite fails, and the legs become dropsical. The causes of this disease are sometimes traceable to hereditary tendencies, but it is brought on or encouraged by eating excessive quantities of vegetable food and drinking extravagantly of water. Once set in it is considered incurable, though its course may end in weeks or years. It is best kept off, or mitigated by abstension from much vegetable food, and by three meals a day of roast beef or mutton, which diet often leads to the most gratifying amelioration. Beer and warm drinks should be avoided, as well as spirits but a regular allowance of good wine is desirable. The premonitory sign being invariably an inordinate desire to eat and drink large quantities, care should be taken to guard against that extravagance.

